# Water Chemistry and Habitat Size Predict Spawning Success in Endangered *Hynobius yangi*: Feeding Ecology and Implications for Urban Wetland Design

**DOI:** 10.3390/ani16091294

**Published:** 2026-04-22

**Authors:** Jeong-Soo Gim, Yoseok Choi, Seoyoon Bae, Kanghui Kim, Suk-Hwan Hong, Mi-Yeon An, Erik Jeppesen, Gea-Jae Joo, Hyunbin Jo

**Affiliations:** 1 Department of Companion Animal Health, Tongmyung University, Busan 48520, Republic of Korea; 2 Department of Biological Science, Pusan National University, Busan 46241, Republic of Korea; 3 Department of Landscape Architecture, Pusan National University, Busan 46241, Republic of Korea; 4 Department of Ecoscience, Aarhus University, 8000 Aarhus, Denmark; 5 Sino-Danish Centre for Education and Research (SDC), University of Chinese Academy of Sciences, Beijing 100190, China; 6 Limnology Laboratory, Department of Biological Sciences and Centre for Ecosystem Research and Implementation, Middle East Technical University, Ankara 06800, Turkey; 7 Institute of Marine Sciences, Middle East Technical University, Mersin 33731, Turkey; 8 Institute for Ecological Research and Pollution Control of Plateau Lakes, School of Ecology and Environmental Science, Yunnan University, Kunming 650504, China

**Keywords:** amphibian extinction risk, habitat restoration, *Hynobius yangi*, diet analysis, metabarcoding, alternative habitats

## Abstract

Urbanization threatens amphibians through habitat loss and fragmentation. The critically endangered *Hynobius yangi*, endemic to Korea, faces severe habitat destruction from urban development. No previous study has simultaneously assessed physicochemical habitat quality and larval feeding ecology across restored and alternative wetlands for this species using fecal DNA metabarcoding. We compared 25 *H. yangi* spawning sites in Sasong New Town through long-term monitoring (April 2021–September 2024; 364 surveys) and fecal DNA metabarcoding (18S V9, COI313, and blocking primers) from 60 larvae. Egg sac abundance showed negative associations with habitat area (r = −0.21), pH (r = −0.23), and conductivity (r = −0.21); however, none retained significance after Bonferroni correction, and each explained only 4–5% of variance, indicating exploratory associations. Associated conditions included area 115.5 ± 16.2 m^2^ (mean ± SE), circularity 44.2 ± 2.4%, pH 7.55 ± 0.10, and conductivity 53.0 ± 2.7 μS/cm. Dietary analysis identified 17 prey taxa. Larvae in alternative areas showed generalist feeding favoring Perlidae and Tubificidae, while restored-area larvae showed specialist patterns dominated by Chironomidae, Nematocera, and Psychodidae. Both habitat types supported *H. yangi* populations. These preliminary findings suggest that appropriately designed alternative areas may complement traditional restoration, pending multi-site validation.

## Simple Summary

The Gori salamander (*Hynobius yangi*) is a critically endangered species found only in South Korea, now threatened by rapid urban development. This study investigated which habitat conditions best support its survival and reproduction in a newly built urban area (Sasong New Town, Busan), comparing actively restored wetlands with alternative habitats that developed naturally alongside urban construction. We found that this salamander was more frequently associated with small, roughly circular water bodies with low pH and low conductivity, and that alternative habitats supported a wider variety of food sources for larvae than restored sites. These findings suggest that conservation strategies should not rely solely on traditional restoration but should also incorporate the creation of small, appropriately designed alternative habitats. Our results provide preliminary guidelines—including exploratory water chemistry associations and habitat shape criteria—that may inform conservation planning for *H. yangi* and potentially other urban-threatened amphibian species, pending multi-site validation.

## 1. Introduction

Urban expansion has accelerated in response to increasing population pressures [1], significantly disrupting natural ecosystems and causing habitat degradation and fragmentation for numerous species [2,3]. Rising public awareness of biodiversity loss has spurred efforts to restore habitats affected by urban development [4,5,6,7].

Amphibians are among the most endangered vertebrates globally, with substantial proportions of species threatened with extinction [8]. Urban development is a primary driver of decline, causing habitat fragmentation, pollution, and altered hydrology that particularly affects species requiring both aquatic and terrestrial environments [9,10].

To evaluate biodiversity and ecosystem recovery in restored areas, various taxa including fish [11], amphibians [12], and birds [13] are used as bioindicators. Amphibians are particularly sensitive indicators given their dual reliance on aquatic and terrestrial environments [14,15], and salamanders are especially vulnerable to water quality disturbances due to their extended aquatic residency [16,17], warranting special conservation attention during large-scale urban development.

In South Korea, the critically endangered *Hynobius yangi* has experienced severe habitat loss due to rapid urban development [18,19], prompting conservation efforts such as relocating individuals to alternative habitats [20]. For successful conservation, it is crucial to identify the key environmental factors influencing reproductive success and to create conditions that closely resemble those of natural habitats [21].

A key indicator of amphibian breeding habitat suitability is oviposition success, measured through egg mass presence and abundance [22]. Factors affecting oviposition vary considerably among species, including canopy cover [23], habitat area [24], and water chemistry [25], necessitating species-specific assessments [26]. Alternative habitat creation—establishing new functional habitats such as drainage channels, retention ponds, and roadside water bodies that spontaneously develop suitable physical and chemical conditions for target species, while acknowledging landscape-level changes rather than recreating historical conditions [27,28,29]—offers a potentially important tool for conservation in heavily modified landscapes.

Physical habitat features alone cannot guarantee ecological functionality [30]; urban development also disrupts food web structure and prey availability, directly affecting species survival and reproduction [31]. Among available dietary assessment methods [32,33], non-invasive fecal analysis is particularly appropriate for endangered species, and its integration with next-generation sequencing (NGS) provides high-resolution identification of prey items that is unattainable through traditional approaches [34,35]. Evaluating both habitat structure and food web dynamics is therefore essential for designing effective compensatory mitigation strategies [36].

Traditional habitat restoration aims to recreate historical conditions through deliberate interventions such as wetland excavation, hydrological modification, and vegetation planting, but this may be insufficient in highly modified urban landscapes where original habitats are irreversibly altered [37]. Alternative habitat creation offers a complementary approach, establishing new functional habitats—such as drainage channels, retention ponds, and roadside water bodies—that spontaneously develop suitable conditions for target species without targeted interventions, thereby acknowledging landscape-level changes rather than reversing them [27,28,29]. However, the relative effectiveness of these approaches for endangered amphibians remains poorly understood, particularly regarding their ability to support stable food webs and reproductive success [38,39].

To evaluate suitable habitat factors, we investigated the characteristics of restored and alternative areas within a new urban development area to examine correlations with egg sac abundance and compare the food web structure between restored and alternative area types where egg sacs were most abundant. Our study had three main objectives: (1) analyzing the characteristics of spawning habitats associated with *H. yangi*’s egg laying behavior, (2) identifying food sources for *H. yangi* larvae using next-generation sequencing (NGS) analysis to observe differences in food sources between larvae in restored and alternative areas, and (3) developing preliminary, evidence-based guidelines for habitat designs that may support both reproductive success and prey availability to inform conservation strategies for this endangered species, with potential transferability to other urban-threatened amphibians pending multi-site validation. Despite advances in amphibian habitat ecology, key knowledge gaps remain: which specific physicochemical parameters best predict *H. yangi* spawning success in urban landscapes, whether alternative habitats support food webs comparable to restored sites, and how larval feeding strategies differ between habitat types. To address these gaps, we formulated the following hypotheses: (1) restored and alternative areas would exhibit different environmental characteristics affecting egg sac abundance, (2) restored areas, shaped by active planting and substrate management, would harbor pollution-tolerant early-successional invertebrates (e.g., Chironomidae), while alternative areas accumulating prey through passive succession would support a broader, more generalist prey community, and (3) *H. yangi* larvae would display habitat-specific feeding strategies reflecting the available prey resources.

## 2. Materials and Methods

### 2.1. Study Area Description and Study Animal

Sasong New Town is a new urban development area completed from 2017 to 2023, covering a total area of 276.6 ha. This area was the primary habitat of *Hynobius yangi* connected to Geumjeongsan Mountain in Busan. During the development process, artificial concrete waterways and catchment areas were installed, and the Dabangcheon and Naesongcheon streams flowing through the center of the development area were modified, causing significant damage to the movement and reproduction of *H. yangi* [40].

The Gori Salamander (*Hynobius yangi* Kim, Min & Matsui, 2003 [41]) is an endemic species found exclusively in Korea. *H. yangi* occurs only in the southern part of Korea and has low mobility, which limits its dispersal ability [42,43]. Therefore, it was designated as a Class 2 endangered wild animal by the Ministry of Environment in 2017 and is currently protected [41,44]. Among the 11 salamander species occurring in Korea, *H. yangi* is the only one classified as endangered [45].

**Figure 1:**
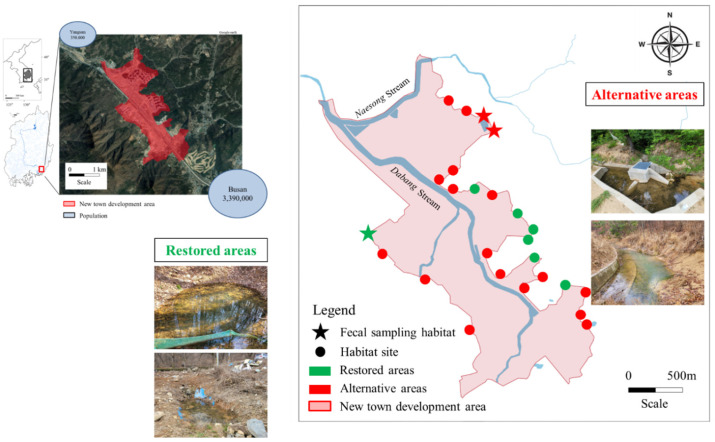
Study sites in Sasong New Town, Busan. The area encompasses the Yangsan stream watershed with its main tributaries (Naesong stream and Dabang stream). Red and green circles represent survey sites, with green indicating restored areas and red indicating alternative areas. Stars (green for restored, red for alternative) mark additional locations where fecal sampling was conducted. The new town development area is outlined in red. Photos show examples of restored and alternative sampling locations. Satellite imagery © Google Maps.

We conducted long-term monitoring of *H. yangi* populations from 23 April 2021 to 10 September 2024, comprising 364 surveys that combined monthly population assessments and weekly habitat monitoring efforts. This extensive 3.5-year study period enabled a comprehensive evaluation of habitat restoration effectiveness and population dynamics across multiple breeding cycles. To evaluate the impact of urban development and the effectiveness of restoration efforts, we classified 25 spawning habitats within the new city development area into two categories: (1) restored areas—areas where active restoration efforts including wetland excavation, hydrological modification, and vegetation planting were implemented to recreate original wetland conditions; detailed standardized documentation of site-specific interventions was not recorded during the study period, which we acknowledge as a limitation, and (2) alternative areas—habitats that emerged alongside urban construction without targeted restoration interventions—such as drainage channels, retention ponds, and roadside water bodies—but which spontaneously developed physical and chemical conditions suitable for *H. yangi*, thereby distinguishing them from both actively restored wetlands and pre-existing natural habitats. This binary classification reflects the operational management categories assigned by the implementing agency; we acknowledge that treating restoration intensity as a continuous variable would provide greater analytical resolution, and that in borderline cases the distinction may be imprecise. Several confounding variables were not systematically controlled, including site age, community assembly time, surrounding land use, human disturbance levels, and distance from source populations; these are identified as limitations and priorities for future research designs [46] (Figure 1). Our study design allowed direct comparison of conservation approaches within a single development project, providing practical insights useful for endangered species management in urban contexts.

### 2.2. Water Quality and Egg Sac Survey

We conducted intensive water quality measurements during the peak spawning season from February to May in 2023 and 2024 for detailed comparative analysis among sites. Variables included water temperature, dissolved oxygen (DO), pH, conductivity, turbidity, and alkalinity. A DO meter (YSI Model 58, Yellow Springs Instruments, Yellow Springs, OH, USA) was used to measure water temperature and DO; conductivity and pH were measured using a conductivity meter (YSI model 152; Yellow Springs Instruments, Yellow Springs, OH, USA) and a pH meter (Orion Model 250A; Orion Research Inc., Boston, MA, USA), respectively. Turbidity was measured using a turbidity meter (Micro 100 turbidimeter, Scientific Inc., Fort Myers, FL, USA), and alkalinity was determined using bromocresol green as an indicator in an acid titration method [47]. Water quality measurements were not conducted at sites where the water had completely dried up.

Egg sac abundance was measured by 12 trained citizen scientists following standardized guidelines for the national distribution survey of endangered wild animals established by the National Institute of Ecology [48,49]. Prior to fieldwork, all participants completed a half-day training session covering egg sac identification, counting protocols, and standardized search procedures. Inter-observer reliability was assessed by having each participant independently count egg sacs at the same site on the same day; counts were subsequently reconciled by the lead researcher to minimize observer bias. Since *H. yangi* lays egg sacs in pairs and the number of eggs in each sac increases with larval growth, we measured spawning ground utilization frequency based on the number of egg sacs present [44,50].

### 2.3. Faeces from H. yangi Larvae Sampling and Pretreatment

All field surveys and sample collection procedures involving the endangered species *H. yangi* were conducted under permit No. 2024-1 issued by the Nakdong River Basin Environmental Office in accordance with the Wildlife Protection and Management Act of Korea. Detailed ethical approval information is provided in Appendix A. From March to June, 2024, we conducted fecal sampling from 127 *H. yangi* larvae across study sites during both daytime (10:00–16:00) and nighttime (20:00–02:00). Quantitative surveys were performed using the line-transect method [51] and the time-constrained method [52]. *H. yangi* larvae (less than 30 mm total length) observed within 1 m on either side of the transect line were carefully identified and temporarily housed in acclimation storage boxes made with garden nets (mesh size: 1 × 1 mm). Of 127 captured larvae, fecal samples were successfully obtained from 60 individuals (47.2%); the remaining 67 either did not produce fecal material within the holding period or produced insufficient material for analysis, a common challenge in fecal sampling of small larval amphibians. To minimize stress and ensure natural excretion, larvae were held on-site for at least six hours based on amphibian excretion times reported by Whiles et al. [53]. These 60 samples were then employed in a comprehensive three-tiered molecular analysis approach. We acknowledge several limitations of this sampling approach: the 6 h holding duration has not been specifically validated for *H. yangi* larvae, though we consider it a reasonable approximation given similarities in body size and feeding rates among small aquatic larvae; larvae were not size-matched between habitat types, which may introduce ontogenetic confounding in dietary comparisons; and multiple larvae from the same site may not represent fully independent dietary observations, as spatial pseudoreplication was not formally addressed. Size-controlled sampling and hierarchical mixed-effects models are recommended for future studies. After this period, fecal material was gently retrieved from the storage boxes, and *H. yangi* larvae were immediately released unharmed at the exact location where they were found. The 60 collected fecal samples were placed in separate 1.5 mL microcentrifuge tubes and transported to the laboratory for refrigerated storage at −80 °C until further analysis. All 60 samples underwent successful DNA extraction, enabling our multi-primer analytical approach described below. The non-invasive fecal sampling approach was essential for studying this critically endangered species while minimizing disturbance to breeding populations.

### 2.4. DNA Analysis

Genomic DNA was extracted from all 60 fecal samples using the DNeasy Blood and Tissue Kit (Qiagen, Hilden, Germany) following the manufacturer’s protocols. We employed a comprehensive three-tiered metabarcoding approach to maximize dietary information and ensure robust taxonomic identification: (1) initial broad taxonomic assessment using 18S V9 primers, (2) detailed species-level identification using COI313 primers, and (3) validation analysis using *H. yangi*-specific blocking primers to minimize host DNA contamination. The 18S V9 primers target a broad taxonomic range, enabling comprehensive prey community assessment, while COI313 primers provide higher taxonomic resolution for species-level identification (Table A1). Additionally, we developed blocking primers specifically targeting *H. yangi* sequences to reduce host DNA amplification bias and enhance prey detection accuracy (Table A2).

PCR amplification, library preparation, and Illumina iSeq sequencing were performed according to established protocols (detailed in Appendix A). For the primary metabarcoding analysis, 36 representative fecal samples (18 from restored areas and 18 from alternative areas) were successfully analyzed using 18S V9 primers. COI-based analysis was completed for 31 of these samples (14 from restored areas and 17 from alternative areas). The lower COI313 success rate (86.1% vs. 100% for 18S V9) most likely reflects the longer COI amplicon amplifying less efficiently from degraded fecal DNA (see Appendix A); primer specificity differences may also have contributed. The slight sample imbalance between habitat types (14 vs. 17) is unlikely to introduce systematic bias but should be considered when comparing diversity metrics. An additional 24 samples (12 from each habitat type) underwent blocking primer analysis using the Block_18SV9 approach to validate findings and detect additional prey taxa while minimizing host DNA interference.

Raw sequences were processed using USEARCH (v11.0.667) with quality filtering, OTU clustering at 97% similarity, and taxonomic assignment via NCBI BLAST+ [2.16.0] searches. Detailed bioinformatics parameters and data processing workflows are provided in Appendix A. OTUs identified as *H. yangi* were considered ‘self-DNA’ and excluded from dietary analyses. The obtained sequences were deposited in the NCBI repository under accession numbers PRJNA1284613 and PRJNA1281337.

### 2.5. Dietary Metrics

The metabarcoding data were presented using two dietary metrics, frequency of occurrence and relative read abundance [54], to indicate overall prey composition detected in the fecal samples of *H. yangi*. The frequency of occurrence (FOO) is the number of samples containing a given prey item. A high FOO value suggests that the prey item is widespread and common in the study area. The relative read abundance (RRA) represents the average of the relative number of reads a prey item occupies in each sample. A high RRA indicates that the prey item is abundant and represented by a large number of reads in the samples. %FOO and %RRA for prey item *i* were calculated as follows:

(1) $$\%{FOO}_{i}\frac{1}{S}\sum_{k=1}^{S}I_{i,k}\times 100\%,$$

(2) $$\%{RRA}_{i}\frac{1}{S}\sum_{k=1}^{S}\frac{n_{i,k}}{\sum_{i=1}^{T}n_{i,k}}\times 100\%,$$

where *T* is the number of prey items, *S* is the number of samples, *I* is an indicator function such that *I_i,k_* = 1 if prey item *i* is present in sample *k*, and 0 otherwise, and *n_i,k_* is the number of reads of prey item *i* in sample *k*.

### 2.6. Costello Method

**Figure A1:**
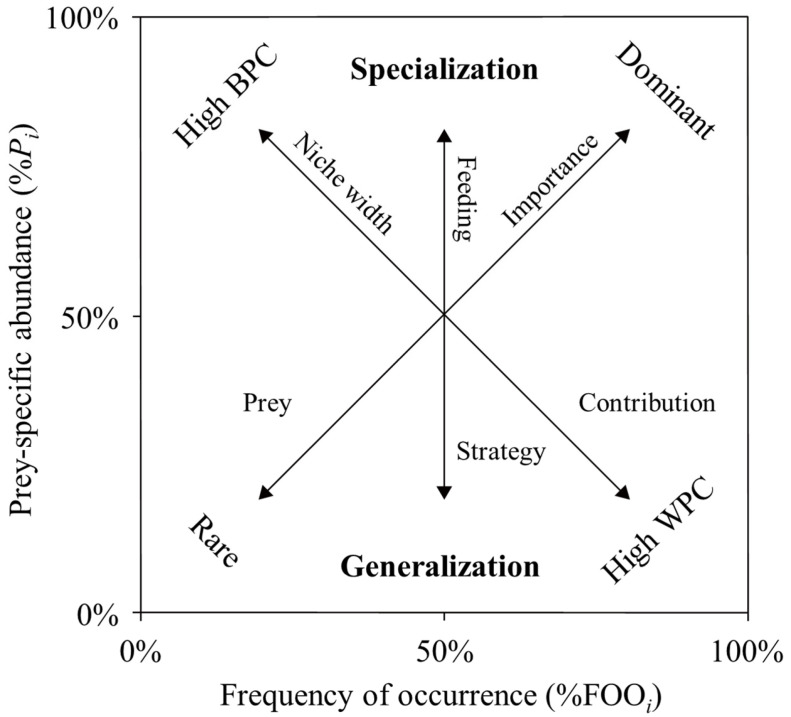
Theoretical diagram of the Costello method. The distribution of the points (each prey item) along the axes provides a graphical interpretation of feeding strategy, prey importance, and niche width contribution (BPC: between-phenotype component; WPC: within-phenotype component).

To examine the dietary characteristics of *H. yangi* larvae graphically, we employed the Costello method [55]. The prey-specific abundance (%*P_i_*) was plotted against %FOO on a two-dimensional graph (Figure A1). To apply this method to the sequencing data, %*P_i_* for prey item *i* was calculated as follows:

(3) $$\%P_{i}=\sum S_{i}/\sum S_{t_{i}}\times 100\%,$$

where *S_i_* is the sum of reads for prey item *i*, and $S_{t_{i}}$ is the sum of reads for samples including prey item *i*. The vertical axis represents a feeding strategy in terms of specialization (i.e., predation concentrated on several prey items) and generalization (i.e., occasional predation on more diverse prey items). The diagonal axis from the upper left to the lower right represents the niche width contribution in terms of the high between-phenotype component (BPC; use of different resources between the individuals) and the high within-phenotype component (WPC; use of various resources within an individual). The diagonal axis from the upper right to the lower left represents the importance of the prey in terms of dominance and rarity.

### 2.7. Selectivity Index and Reference Data

To examine the prey selection of *H. yangi* larvae for the available food in the habitat, we used Jacobs’s selectivity index [56]. Preferences for fish and benthic invertebrates were calculated separately, based on the %RRA of the prey items and field survey data. For benthic invertebrate abundance data, we utilized comprehensive survey results from the final report by Kyungdong Engineering [49], which documented invertebrate communities surveyed using standardized field collection methods, including dip net sampling, kick-net techniques, and systematic substrate sampling across habitat types within our study area. These standardized survey data provided the reference baseline for calculating prey availability and selectivity indices in our analyses. Prey items were analyzed at the family level due to the identification limit of the field survey. The Jacobs’ index *D* for food item *i* was calculated as follows:

(4) $$D_{i}=\frac{r_{i}-p_{i}}{r_{i}+p_{i}-2r_{i}p_{i}},$$

where *r_i_* is the RRA of prey item *i*, and *p_i_* is the ratio of prey item *i* to the total number of fish or benthic invertebrates surveyed in the field. The index ranges from −1 (negative selection) to 1 (positive selection), and a value of 0 means no preference or avoidance.

### 2.8. Statistical Analysis

Since previous studies have examined salamander oviposition preferences and terrestrial habitat extent [57] but not the circularity of aquatic habitats, we included habitat circularity as an exploratory variable. Ecologically, circular water bodies may provide more uniform shoreline-to-open-water ratios, potentially reducing exposure to terrestrial predators and creating more homogeneous thermal and microhabitat conditions. This analysis was hypothesis-generating rather than hypothesis-testing, and circular associations should be interpreted accordingly. To measure the area of the spawning ground, we used ImageJ software (version 1.53k, National Institutes of Health, Bethesda, MD, USA) based on drawings of the spawning grounds. To evaluate the circularity of the lake, we processed the image using Python [3.12.8] and OpenCV [4.11.0]. The boundary contour was extracted, and the minimum enclosing circle was computed to determine the circularity similarity using the following formula:

(5) Similarity(%)=(Contour Area/Circle Area)×100

The contour was detected using OpenCV’s cv2.findContours function, and the minimum enclosing circle was calculated using cv2.minEnclosingCircle [58]. The analysis was conducted in Python [3.12.8] [59] using OpenCV [4.11.0] [58] and NumPy [60].

Environmental variables, including water quality variables (water temperature (°C), pH, dissolved oxygen (mg/L and %), conductivity (μS/cm), turbidity (NTU), and alkalinity (mg/L as CaCO_3_)) and habitat characteristics (area (m^2^) and circularity), were standardized using Z-score transformation to allow direct comparisons across different measurement units. The Z-score for each variable was calculated as:

(6) $$Z=\frac{X-\mu }{\sigma }$$

where *X* represents the raw value, μ is the mean, and σ is the standard deviation of the variable. To determine the correlation between egg sac and spawning site characteristics and water quality, principal component analysis (PCA) was performed using R version 4.0.5.

Statistical analyses were performed using Paleontological Statistics (PAST) 4.05 (Natural History Museum, Oslo, Norway). Rarefaction analysis was performed to determine the sample size and the number of reads required to represent sufficient OTU richness. All statistical tests were performed with an α = 0.05 significance level. To evaluate differences in prey community composition between habitat types, permutational multivariate analysis of variance (PERMANOVA; adonis2 function, vegan package v2.6, R v4.0.5) was applied separately to frequency of occurrence (FOO) and relative read abundance (RRA) matrices for each primer set (18S V9, COI313, and Block_18SV9). Jaccard dissimilarity was used for binary FOO matrices and Bray–Curtis dissimilarity for RRA matrices, each with 9999 permutations.

We note several analytical limitations. Water quality measurements were conducted only in 2023–2024, whereas egg sac data span 2021–2024; we assumed physicochemical properties were relatively stable across years, an assumption requiring future investigation. Second, sites along the same stream system may not be fully spatially independent; spatial autocorrelation was not formally tested, representing an additional limitation of the correlational analyses. Individual Pearson correlations should be interpreted with these limitations in mind.

## 3. Results

### 3.1. Water Quality Parameters and Habitat Characteristics

**Figure 2:**
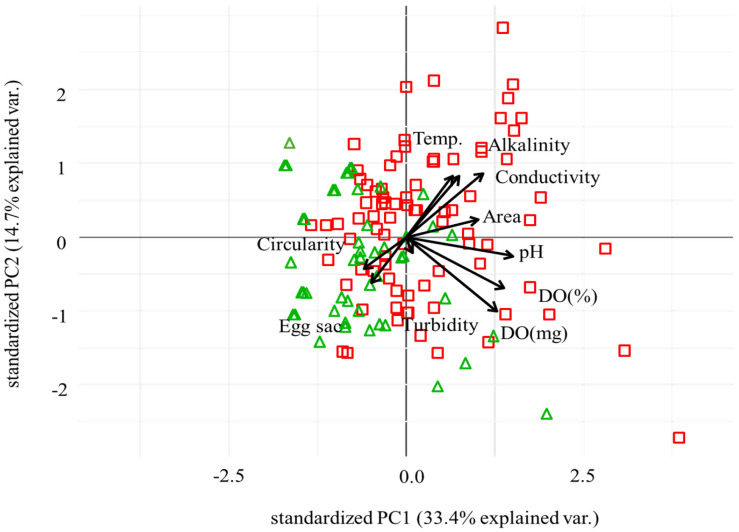
Principal component analysis (PCA) of ten variables (egg sac, dissolved oxygen (DO) in % and mg/L, pH, alkalinity, turbidity, conductivity, temperature (Temp.), area, and circularity). Restored areas are represented by green triangles, alternative areas by red squares.

We analyzed nine environmental variables (temperature, pH, DO (mg/L), DO (%), conductivity, alkalinity, turbidity, area, circularity) using PCA to evaluate their correlations with egg sac abundance (Figure 2). In the ordination, 33.3% of the variation was explained by axis 1 (PC1), while axis 2 (PC2) explained 14.6% (total 48.0% of variance) in the biplot. The remaining 52% of variance was not captured by these two axes, indicating that substantial environmental variation lies beyond PC1 and PC2; the biplot should be interpreted with this limitation in mind; future studies should consider examining additional PC axes or alternative multivariate approaches to capture remaining environmental variation. Area and pH showed relatively strong positive correlations with PC1, while alkalinity and temperature showed relatively strong negative correlations with PC2.

Egg sac abundance showed negative correlations with conductivity (r = −0.211, *p* = 0.018), area (r = −0.205, *p* = 0.022), and pH (r = −0.232, *p* = 0.009) prior to correction for multiple comparisons (Table 1). Following Bonferroni correction for nine simultaneous tests, none of these correlations retained statistical significance (corrected *p*: pH = 0.082, conductivity = 0.163, area = 0.196), and all should therefore be interpreted as exploratory associations. These correlations individually account for only 4–5% of the variance in egg sac abundance, and post hoc power analysis indicated insufficient statistical power for all variables tested (maximum power = 0.747 for pH), suggesting that the current sample size may have been inadequate to detect all biologically meaningful relationships. VIF analysis confirmed severe collinearity between DO (mg/L) and DO (%) (VIF > 36), reflecting their structural redundancy as duplicate measures of the same parameter; all remaining variables showed acceptable VIF values (<6), including area (VIF = 1.37) and circularity (VIF = 1.17). In the biplot, each shape represents different habitat types: restored areas (triangles) and alternative areas (squares). Physicochemical properties of restored areas (triangles) appeared to cluster on the left side of the biplot, where relatively positive correlations with egg sac abundance and circularity and negative correlations with pH and area were observed (Figure 2). Physicochemical properties of alternative areas (squares) spanned the entire range of the biplot, with no discernible clustering patterns.

Sites positioned in the positive direction along the axis with egg sac abundance showed the following mean values: pH (7.55 ± 0.10), electrical conductivity (53.0 ± 2.7 μS/cm), habitat area (115.5 ± 16.2 m^2^), and circularity (44.2 ± 2.4%) (Table 2). These are descriptive summaries from PCA-selected sites and should not be interpreted as statistically validated target values.

### 3.2. Prey Community Composition Across Habitat Types: Multi-Primer Metabarcoding Results

#### 3.2.1. Broad Taxonomic Assessment Using 18S V9 Primers

DNA metabarcoding analysis using 18S V9 primers generated 6,015,215 paired-end reads from 36 samples. After quality filtering, 5,468,989 sequences were obtained. Through quality filtering with a relative read count threshold, 805 OTUs remained, resulting in 40 taxa after taxonomic assignment (Table A3).

#### 3.2.2. Species-Level Resolution Using COI313 Primers

DNA metabarcoding analysis using COI313 primers generated 2,486,471 paired-end reads from 31 samples. After quality filtering, 1,636,546 sequences were obtained. Through quality filtering with a relative read count threshold, 268 OTUs remained, resulting in 17 taxa after taxonomic assignment (Table A4).

### 3.3. Habitat-Specific Dietary Patterns and Community Structure

#### 3.3.1. Primary Dietary Assessment: 18S V9 Results Across Habitat Types

**Figure 3:**
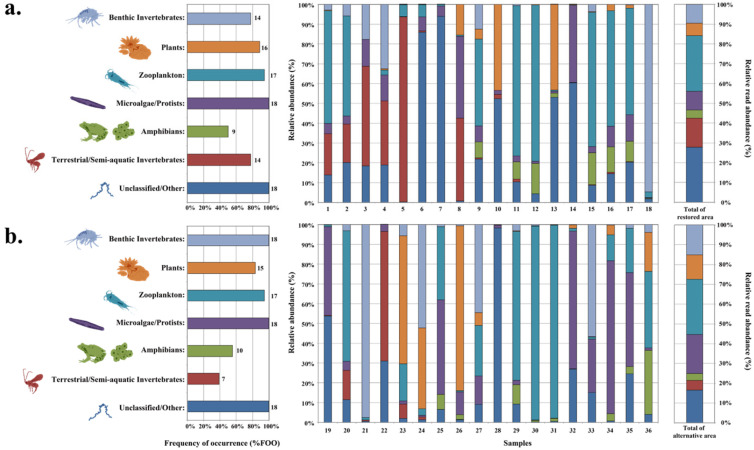
Prey composition analysis using 18SV9 primers comparing restored ((**a**); n = 18) and alternative ((**b**); n = 18) habitats. Left panels show frequency of occurrence (%FOO) with sample numbers indicated at bar edges. Right panels show relative read abundance (%RRA) for each prey category using the same color scheme. PERMANOVA results for habitat-type differences are reported in the Results text.

The dietary metrics FOO and RRA revealed differences in prey composition between restored and alternative areas (Figure 3). The Unclassified/Other group exhibited the highest frequency in both areas (100.0%, 18/18 samples in each area), although its RRA value was lower in alternative areas compared to restored areas. Similarly, the Microalgae/Protists group was present in all samples (100%, 18/18 samples in each area) but showed higher RRA values in alternative areas.

The Benthic Invertebrates group displayed an increase in both FOO (77.8% to 100%) and RRA values in alternative areas. Conversely, the Terrestrial/Semi-aquatic Invertebrates group showed a decrease in both FOO (77.8% to 38.9%) and RRA values in alternative areas. Zooplankton represented the most abundant DNA reads in both restored areas (28.1%) and alternative areas (27.9%), with consistent FOO values (94.4%, 17/18 samples in both areas). The remaining taxa showed similar patterns across both habitat types. PERMANOVA indicated a marginal but non-significant difference between habitat types in FOO (F = 2.919, R^2^ = 0.079, *p* = 0.065) and a non-significant difference in RRA (F = 0.948, R^2^ = 0.027, *p* = 0.459) based on 18S V9 analysis.

#### 3.3.2. Enhanced Species Resolution: COI313 Analysis of Habitat-Specific Prey

**Figure 4:**
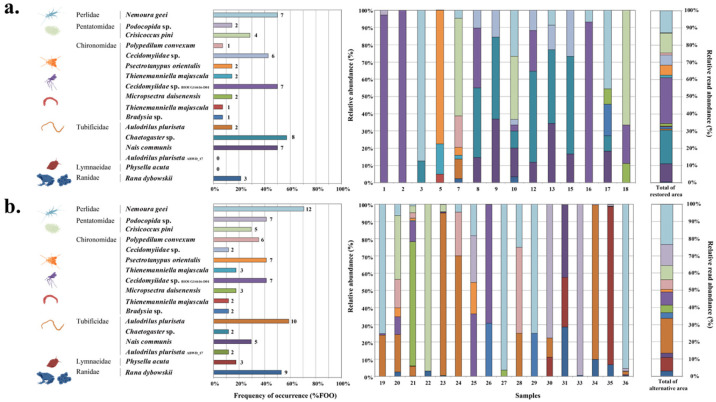
Prey composition analysis using COI313 primers comparing restored ((**a**); n = 14) and alternative ((**b**); n = 17) habitats. Left panels show frequency of occurrence (%FOO) with sample numbers indicated at bar edges. Right panels show relative read abundance (%RRA) for each prey taxon using the same color scheme. PERMANOVA results for habitat-type differences are reported in the Results text.

COI313 primer analysis further differentiated the prey composition between the two habitat types (Figure 4). Taxa in the alternative areas generally exhibited higher FOO values and greater diversity. Notably, Perlidae increased in both FOO (50.0%, 7/14 samples, to 70.6%, 12/17 samples) and RRA values in alternative areas. Similar increases were observed for *Aulodrilus pluriseta* (14.3% to 58.8% FOO) and Ranidae (21.4% to 52.9% FOO). *Aulodrilus pluriseta* ABWD_17 (11.8% FOO) and *Physella acuta* (17.6% FOO) were detected exclusively in alternative areas. In contrast, *Cecidomyiidae* sp. maintained similar FOO values between areas but showed decreased RRA values in alternative areas. PERMANOVA confirmed statistically significant differences between habitat types in both FOO (F = 3.198, R^2^ = 0.099, *p* = 0.003) and RRA (F = 3.329, R^2^ = 0.103, *p* = 0.002) based on COI313 analysis.

#### 3.3.3. Validation Analysis Using Blocking Primers

**Figure 5:**
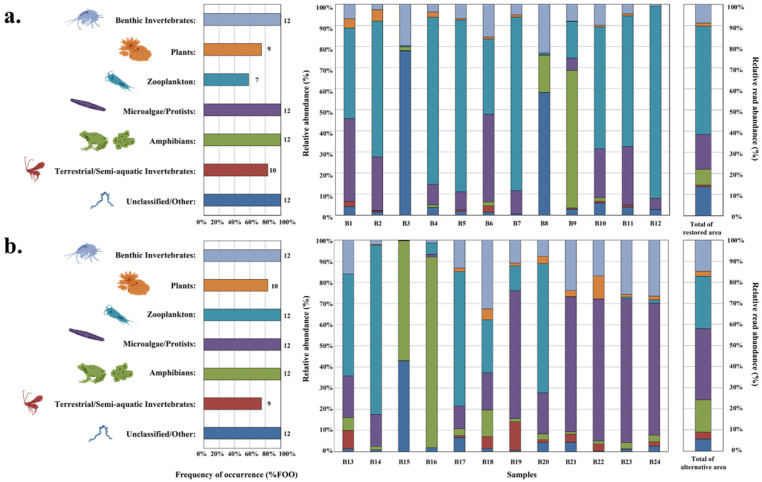
Prey composition analysis using Block_18SV9 primers comparing restored ((**a**); n = 12) and alternative ((**b**); n = 12) habitats. Left panels show frequency of occurrence (%FOO) with sample numbers indicated at bar edges. Right panels show relative read abundance (%RRA) for each prey category using the same color scheme. PERMANOVA results for habitat-type differences are reported in the Results text.

The blocking primer analysis (Figure 5) confirmed our main findings, with alternative areas consistently showing higher prey diversity than restored areas. The Block_18SV9 approach successfully reduced host DNA interference while maintaining prey detection accuracy, revealing 31 taxa in alternative areas compared to 28 in restored areas. This validation analysis demonstrated concordance with both 18S V9 and COI313 results, supporting the robustness of the observed habitat-specific dietary patterns. PERMANOVA indicated a marginal but non-significant difference in FOO (F = 3.357, R^2^ = 0.132, *p* = 0.071) and a non-significant difference in RRA (F = 2.127, R^2^ = 0.088, *p* = 0.105) based on Block_18SV9 analysis.

### 3.4. Habitat-Specific Feeding Strategies: Generalist vs. Specialist Patterns

**Figure 6:**
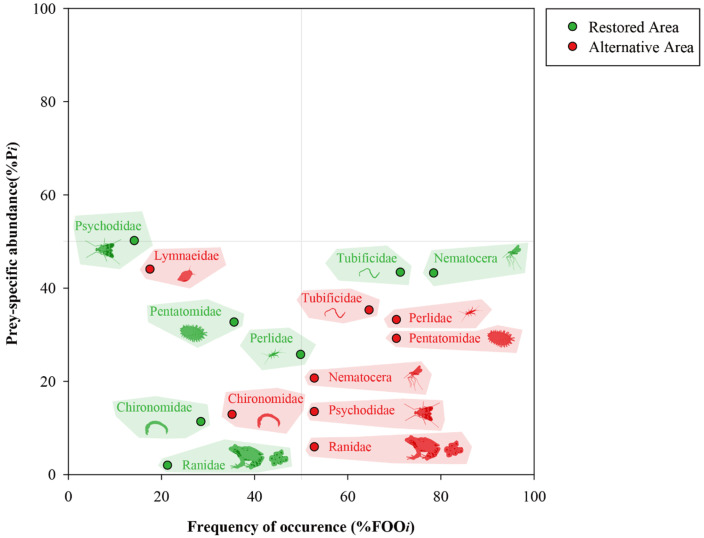
Costello diagram analysis of *Hynobius yangi* feeding habits. Points represent prey items detected in fecal samples (green: restored areas; red: alternative areas). Point distribution provides a graphical interpretation of feeding strategy, prey importance, and niche width contribution.

The Costello diagram comparing prey families between restored and alternative areas revealed distinct feeding patterns of *H. yangi* larvae across habitat types (Figure 6). In restored areas, Psychodidae, Tubificidae, and Nematocera families were positioned in the upper middle region (%FOO between 10–70%, %*P_i_* between 42 and 50%), indicating their significant contribution to the diet when encountered. Pentatomatidae showed moderate occurrence and abundance (%FOO = 35.7%, %*P_i_* = 32.6%), while Chironomidae exhibited lower specific abundance despite similar occurrence (%FOO = 28.6%, %*P_i_* = 11.2%). Perlidae appeared with moderate frequency but lower abundance (%FOO = 50.0%, %*P_i_* = 25.6%), and Ranidae showed minimal contribution to the diet (%FOO = 21.4%, %*P_i_* < 5%).

In contrast, alternative areas displayed a different pattern, with most families positioned in the middle-right portion of the diagram, indicating generally higher occurrence frequencies and suggesting a more generalist feeding strategy.

### 3.5. Prey Selectivity Analysis

**Figure 7:**
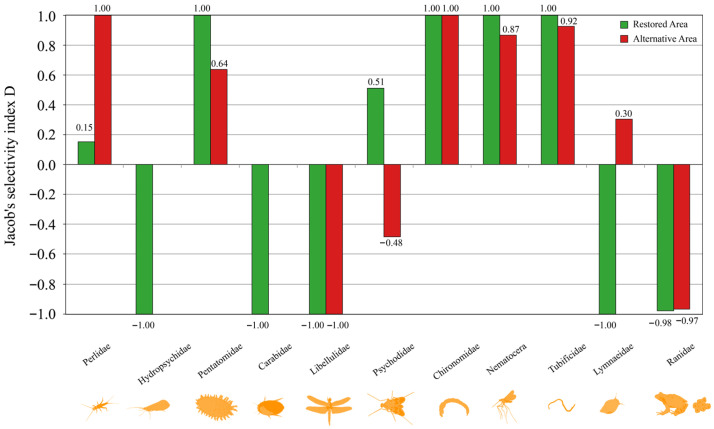
Prey selectivity analysis of *Hynobius yangi* comparing restored (green) and alternative (red) habitats. Analysis based on relative read abundance (RRA) of prey items in fecal samples compared to field survey data. Prey items were analyzed at the family level. Jacobs’ selectivity index ranges from −1 (negative selection) to +1 (positive selection), with 0 indicating no preference.

We calculated the selectivity of *H. yangi* larvae for prey items (Figure 7) based on fecal DNA metabarcoding and field survey data across both restored and alternative areas. In restored areas, *H. yangi* larvae showed strong positive selection (D = 1.00) for Pentatomatidae, Chironomidae, Nematocera, and Psychodidae, moderate positive selection (D = 0.51) for Psychodidae, slight positive selection (D = 0.15) for Perlidae, and strong negative selection for Hydropsychidae (D = −1.00), Carabidae (D = −1.00), Libellulidae (D = −1.00), Tubificidae (D = −1.00), and Ranidae (D = −0.98).

In alternative areas, the selectivity pattern was notably different, with strongest positive selection for Perlidae (D = 1.00), Chironomidae (D = 1.00), and Nematocera (D = 1.00), strong positive selection for Pentatomatidae (D = 0.64) and Tubificidae (D = 0.92), moderate positive selection for Lymnaeidae (D = 0.30), and strong negative selection for Carabidae (D = −1.00), Libellulidae (D = −0.48), and Ranidae (D = −0.97).

Our results suggest that habitat restoration efforts altered the feeding selectivity of *H. yangi* larvae, with increased preference for Perlidae and Tubificidae and decreased avoidance of Lymnaeidae in alternative areas compared to restored areas.

## 4. Discussion

Our study identified physicochemical indices associated with *H. yangi* spawning site selection and characterized larval dietary differences between restored and alternative areas using non-invasive fecal DNA metabarcoding. Egg sac abundance was associated with low pH, low conductivity, and smaller habitat size, while alternative areas supported greater dietary diversity than restored areas [30,61].

Our findings add to the growing evidence that urban amphibian conservation requires nuanced approaches beyond traditional restoration paradigms [43]. The success of alternative area creation challenges the assumption that only historical habitat reconstruction can support endangered species, particularly in landscapes where original conditions are irreversibly altered [62]. This has significant implications for conservation planning in rapidly urbanizing regions, particularly where land availability for restoration is limited and alternative approaches may provide more feasible conservation solutions [63].

### 4.1. Habitat Characteristics and Spawning Site Preferences

We found that egg sac abundance was associated with areas near-circular in shape, with low electrical conductivity and pH, and small in size (Table 1; Figure 2). While previous studies, such as Vági (2013) [64], have reported that larger habitats positively influence amphibian reproduction, our findings suggest that smaller and near-circular areas tend to be associated with higher egg sac abundance in *H. yangi* spawning. This might be attributed to lower predator densities, reduced competition, and more stable microenvironmental conditions in smaller water bodies [24]. This association with smaller, circular areas is consistent with broader patterns observed in pond-breeding salamanders, where edge effects and predation pressure may influence breeding site selection [65]. Circular habitats may provide optimal thermal regimes and reduced exposure to terrestrial predators, factors critical for egg survival in urban environments where natural refugia are scarce [66]. The association with smaller water bodies may also be partly explained by fish predation pressure, as larger habitats typically support fish populations that prey on salamander eggs and larvae, while *H. yangi* appears particularly vulnerable to such predation compared to other salamander species [50,67].

The negative correlations (each explaining only 4–5% of variance, and none retaining significance following Bonferroni correction) suggest a tentative association between *H. yangi* egg sac abundance and smaller, more acidic environments with lower ionic concentrations. These should be regarded as exploratory associations only, as alternative explanations including differential egg survival, unmeasured confounders, and historical colonization cannot be excluded, and post hoc power analysis indicated that the current sample size was insufficient to reliably detect effects of this magnitude. We propose as a testable hypothesis that concrete infrastructure may partly elevate pH through calcium carbonate leaching [68,69]; we further hypothesize that lower pH and conductivity may reduce osmoregulatory stress and slow organic matter decomposition, improving egg survival [70,71]. Both mechanisms require direct empirical validation, including pH measurements near versus away from concrete structures, temporal monitoring post-construction, and comparison with pH values from natural undisturbed *H. yangi* habitats.

Notably, some alternative areas that shared critical environmental features with restored areas also supported higher spawning activity (Figure 2), suggesting that, when properly managed, alternative areas can function similarly to restored sites in supporting *H. yangi* reproduction [72]. This observation highlights the potential for habitat creation to support *H. yangi* populations when specific environmental factors are carefully managed, particularly regarding placement away from concrete structures or incorporation of buffering systems to maintain appropriate pH levels.

### 4.2. Dietary Diversity and Feeding Ecology

The integration of habitat structure and food web analyses represents a crucial advancement in restoration assessment as physical habitat suitability alone does not guarantee ecological functionality [73]. Urban environments often exhibit simplified food webs and altered prey communities, making dietary analysis essential for evaluating whether created areas can support complete life cycles [74].

The dietary analysis of *H. yangi* larvae revealed notable differences in prey composition between restored and alternative areas (summarized in Section 3.4 and Section 3.5), suggesting habitat-specific feeding patterns. Chironomidae and Psychodidae dominated in restored areas, while Perlidae and Tubificidae dominated in alternative areas. While the Unclassified/Other group was universally present in both areas, its lower RRA in alternative areas indicates a shift toward greater dietary diversity. Microalgae/Protists showed higher RRA values in alternative areas, potentially reflecting improved aquatic primary productivity [75]. The increased presence of Benthic Invertebrates in alternative areas (FOO from 77.8% to 100%) suggests successful community establishment following restoration efforts [76], while the decreased representation of Terrestrial/Semi-aquatic Invertebrates (FOO from 77.8% to 38.9%) may indicate habitat-specific resource utilization [77].

The COI313 primer analysis revealed even more pronounced differences, with taxa in alternative areas showing generally higher occurrence and diversity. The increased presence of Perlidae, *Aulodrilus pluriseta*, and Ranidae in alternative areas likely reflects improved habitat conditions [78,79], while the exclusive detection of certain species in alternative areas emphasizes the development of unique ecological niches [80]. These findings collectively suggest that alternative areas were associated with greater prey diversity for *H. yangi* larvae, a pattern statistically supported by COI313-based PERMANOVA (FOO: *p* = 0.003; RRA: *p* = 0.002).

The two metabarcoding approaches proved complementary: 18S V9 achieved higher amplification success (100% vs. 86.1% for COI313) and broader taxonomic coverage, while COI313 provided finer species-level resolution for certain invertebrate prey items [81,82,83]. Their combined use provided a more comprehensive understanding of *H. yangi* larvae’s diet across different habitats than either marker alone.

To address potential host DNA contamination bias [84], blocking primers targeting *H. yangi* sequences were applied to 24 samples (12 per habitat type). The Block_18SV9 results (Figure 5, Table A5) showed consistent patterns with primary 18S V9 and COI313 analyses, confirming greater prey diversity in alternative areas regardless of analytical technique (Table A6). To formally evaluate these differences, PERMANOVA was applied to FOO and RRA matrices for all three primer sets. For 18S V9 (n = 36), habitat type explained a marginal but non-significant proportion of variation in FOO (F = 2.919, R^2^ = 0.079, *p* = 0.065) and a non-significant proportion in RRA (F = 0.948, R^2^ = 0.027, *p* = 0.459). For COI313 (n = 31), habitat type significantly differentiated both FOO (F = 3.198, R^2^ = 0.099, *p* = 0.003) and RRA (F = 3.329, R^2^ = 0.103, *p* = 0.002) matrices. For Block_18SV9 (n = 24), results were marginal for FOO (F = 3.357, R^2^ = 0.132, *p* = 0.071) and non-significant for RRA (F = 2.127, R^2^ = 0.088, *p* = 0.105). Collectively, these results indicate that habitat-associated differences in prey community composition were statistically supported by COI313 analysis, while 18S V9 and Block_18SV9 showed consistent directional trends that did not reach conventional significance thresholds.

### 4.3. Feeding Strategy and Prey Selectivity

The feeding ecology of *H. yangi* larvae showed distinct pattern differences between restored and alternative areas, suggesting habitat-specific prey preferences and resource utilization. The Costello diagram analysis revealed that in restored areas, larvae exhibited a more specialized feeding strategy, with Psychodidae, Tubificidae, and Nematocera families representing dominant prey items when encountered. This contrasts with alternative areas, where most prey families were positioned in the middle-right part of the diagram, indicating a more generalist feeding strategy with higher prey occurrence frequencies.

The selectivity analysis further supported habitat-specific feeding patterns [85]: restored-area larvae strongly preferred Chironomidae, Nematocera, and Psychodidae while avoiding Tubificidae, whereas alternative-area larvae preferred Perlidae and showed increased selection for Tubificidae. The generalist feeding pattern in alternative areas can be interpreted in two contrasting ways—richer prey diversity or prey scarcity forcing opportunistic feeding [86]—and whether it reflects resource abundance or limitation cannot be resolved without direct prey availability measurements. PERMANOVA results support the existence of habitat-specific prey community differences at the multivariate level (COI313: *p* < 0.01), though the ecological mechanism underlying the observed generalist strategy in alternative areas requires further investigation. Habitat type thus appears to shape not only prey community composition but also larval foraging flexibility [87].

The increased selection for Perlidae (D = 1.00) in alternative areas versus restored areas (D = 0.15) is particularly noteworthy, as stonefly larvae are well-established bioindicators of good water quality [88]. This contrast suggests that passive succession in alternative areas may have fostered water quality conditions favorable to pollution-sensitive taxa, while the dominance of Chironomidae and Psychodidae in restored areas may reflect organic enrichment associated with construction disturbance—consistent with the known tolerance of these groups to degraded conditions. Direct benthic invertebrate surveys and water quality indices (e.g., EPT richness, BMWP scores) would be required to confirm this interpretation. These findings align with optimal foraging theory, which predicts that predators modify feeding strategies based on prey availability, accessibility, and nutritional value [55].

### 4.4. Conservation Implications and Management Recommendations

Our findings have important implications for the conservation of *H. yangi* and the design of effective habitat restoration strategies. The association with smaller, circular areas with specific water chemistry parameters suggests that restoration efforts should prioritize creating multiple small-scale wetlands rather than fewer large ones. The conditions associated with higher egg sac abundance (pH 7.55 ± 0.10, conductivity 53.0 ± 2.7 μS/cm, area 115.5 ± 16.2 m^2^, circularity 44.2 ± 2.4%) may serve as preliminary reference targets for habitat design, though these derive from weak correlations at a single site and should be treated as hypothesis-generating guidelines rather than established criteria [89]. We tentatively suggest that future alternative habitat creation may benefit from: (1) placement away from concrete infrastructure, (2) design of multiple small water bodies to limit fish colonization, and (3) measures to promote benthic invertebrate establishment. These suggestions require adaptive management and multi-site validation before broader application.

The greater prey diversity observed in alternative areas is associated with broader dietary breadth, though whether this reflects superior prey resources or opportunistic feeding under resource scarcity cannot be determined without direct prey availability assessments. Whether this dietary breadth translates to improved larval growth, survival, or recruitment similarly cannot be determined from dietary composition data alone—demographic data such as growth rates and metamorphosis success would be required [86]. Conservation strategies should therefore consider both habitat types as complementary components of a landscape-scale approach [90].

Furthermore, the shift from specialist to generalist feeding strategies in alternative areas may indicate increased ecosystem resilience as generalist feeding can buffer against prey fluctuations and environmental changes [91]. However, the ecological consequences of this dietary shift require further investigation to fully understand its implications for larval growth, development, and survival [86]. At the same time, our comprehensive 3.5-year monitoring program provides valuable long-term insights into *H. yangi* habitat requirements and feeding ecology, the intensive dietary analysis was concentrated during peak breeding seasons, which may not capture seasonal variations in prey availability and dietary preferences throughout the entire annual cycle [20]. Additionally, the focus on larval diet may not reflect the complete nutritional requirements throughout the species’ life cycle. Long-term monitoring is needed to assess the stability of created areas and their continued suitability as urban landscapes evolve [92]. Future research should also investigate genetic connectivity between populations using different area types to ensure long-term population viability [93], examine the cost-effectiveness of alternative area creation compared to traditional restoration approaches [94], and develop standardized protocols for urban amphibian habitat design [43]. Furthermore, future research should investigate the relationship between dietary diversity and larval fitness parameters, such as growth rates and survival, with metamorphosis. Long-term monitoring of both restored and alternative areas would provide insights into the sustainability of observed patterns and the long-term effectiveness of different restoration approaches [95].

## 5. Conclusions

This study demonstrates that both restored and alternative areas can support *H. yangi* populations within urban development contexts, though with distinct ecological characteristics. *H. yangi* egg sac abundance showed negative associations with habitat area, pH, and conductivity, and a positive association with near-circular shape, though none of these correlations retained statistical significance following Bonferroni correction for multiple comparisons. Each variable individually explained only 4–5% of variance, and post hoc power analysis indicated insufficient statistical power across all variables tested, indicating that these findings should be regarded as exploratory associations requiring confirmation in larger-scale studies. These findings highlight the importance of water chemistry and morphology management in conservation planning, including the potential need to avoid concrete infrastructure (a mechanism requiring empirical validation). Fecal DNA metabarcoding revealed that alternative areas were associated with greater prey diversity and generalist feeding strategies in *H. yangi* larvae, while larvae in restored areas exhibited more specialized dietary patterns. PERMANOVA confirmed statistically significant habitat-associated differences in prey community composition based on COI313 analysis, with consistent directional trends observed across 18S V9 and Block_18SV9 analyses, supporting the utility of non-invasive fecal metabarcoding for dietary assessment of critically endangered species. Collectively, our findings suggest that appropriately designed alternative habitats may complement traditional restoration in urbanized landscapes, though this inference is based on correlational data from a single site. We tentatively suggest integrated landscape-scale strategies combining both approaches, with a focus on small, near-circular wetlands associated with lower pH (~7.55) and conductivity (~53.0 μS/cm) and situated away from concrete infrastructure. These suggestions require replication and experimental validation before broader application. They provide a preliminary basis for hypothesis-driven conservation planning for *H. yangi* and may offer transferable insights for other urban-threatened amphibian species pending multi-site validation.

## Figures and Tables

**Table 1:** Pearson correlation coefficients and uncorrected *p*-values between variables. Significance levels prior to Bonferroni correction: * *p* < 0.05 and ** *p* < 0.01. Following Bonferroni correction for nine simultaneous comparisons, no correlations with egg sac abundance remained statistically significant (refer to Section 3.1).

	Egg Sac	Temp.	pH	DO (mg/L)	DO (%)	Conductivity	Alkalinity	Turbidity	Area	Circularity
Egg sac	1	−0.158 0.079	−0.232 ** 0.009	−0.041 0.647	−0.082 0.363	0.211 * 0.018	−0.125 0.166	0.028 0.759	−0.205 * 0.022	−0.061 0.499
Temp	−0.158 0.079	1	0.224 * 0.012	−0.048 0.599	0.275 ** 0.002	0.445 ** 0.000	0.057 0.528	0.026 0.773	0.187 * 0.037	−0.054 0.546
pH	−0.232 ** 0.009	0.224 * 0.012	1	0.707 ** 0.000	0.736 ** 0.000	0.466 ** 0.000	0.292 ** 0.001	0.051 0.575	0.472 ** 0.000	−0.206 * 0.021
DO (mg/L)	−0.041 0.647	−0.048 0.599	0.707 ** 0.000	1	0.932 ** 0.000	0.213 * 0.017	0.107 0.235	0.019 0.834	0.278 ** 0.002	−0.142 0.115
DO (%)	−0.082 0.363	0.275 ** 0.002	0.736 ** 0.000	0.932 ** 0.000	1	0.335 ** 0.000	0.135 0.135	0.025 0.783	0.300 ** 0.001	−0.134 0.136
Conductivity.	−0.211 * 0.018	0.445 ** 0.000	0.466 ** 0.000	0.213 * 0.017	0.335 ** 0.000	1	0.441 ** 0.000	−0.047 0.605	0.249 ** 0.005	−0.240 ** 0.007
Alkalinity	−0.125 0.166	0.057 0.528	0.292 ** 0.001	0.107 0.235	0.135 0.135	0.441 ** 0.000	1	0.128 0.155	0.165 0.066	−0.265 ** 0.003
Turbidity	0.028 0.759	0.026 0.773	0.051 0.575	0.019 0.834	0.025 0.783	−0.047 0.605	0.128 0.155	1	−0.022 0.804	0.099 0.270
Area	−0.205 * 0.022	0.187 * 0.037	0.472 ** 0.000	0.278 ** 0.002	0.300 ** 0.001	0.249 ** 0.005	0.165 0.066	−0.022 0.804	1	−0.243 ** 0.006
Circularity	−0.061 0.499	−0.054 0.546	−0.206 * 0.021	−0.142 0.115	−0.134 0.136	−0.240 ** 0.007	−0.265 ** 0.003	0.099 0.270	−0.243 ** 0.006	1

**Table 2:** Descriptive summary of water quality parameters for sites positioned in the positive direction along the egg sac abundance axis in the PCA biplot (Figure 2). These values represent conditions associated with higher egg sac abundance and should not be interpreted as statistically validated optima. Values are presented as mean ± SE (standard error) and 95% confidence interval.

Parameters	Average	SE	95% CI
Temp	13.0	0.8	[11.43, 14.57]
pH	7.55	0.10	[7.35, 7.75]
DO (mg/L)	11.1	0.3	[10.51, 11.69]
DO (%)	105.0	2.5	[100.1, 109.9]
Conductivity	53.0	2.7	[47.71, 58.29]
Alkalinity	18.5	1.1	[16.34, 20.66]
Turbidity	8.6	1.6	[5.46, 11.74]
Area	115.5	16.2	[83.75, 147.25]
Circularity	44.2	2.4	[39.5, 48.9]

## Data Availability

The original datasets analyzed in the study are publicly available. This data can be found here: NCBI repository under accession number: PRJNA1284613 and PRJNA1281337.

## Appendix A

### Appendix A.1. DNA Extraction and Amplification

Faecal samples from *Hynobius yangi* larvae were homogenized using sterilized homogenizer pestles, and genomic DNA was extracted using the DNeasy Blood and Tissue Kit (Qiagen, Hilden, Germany) according to the manufacturer’s instructions. The extracted DNA samples were stored at −20 °C.

To comprehensively identify food sources in the faecal samples, we employed a two-tiered NGS approach using two different universal eukaryotic markers. Two consecutive PCR steps were performed for the next-generation sequencing (NGS) process, with both negative and positive controls employed throughout the entire PCR process.

First, PCR amplification was performed using primers targeting the V9 region of 18S rRNA (18SV9_F and 18SV9_R; Table A1) to capture a broad taxonomic overview. Subsequently, a second set of first PCR amplification was conducted using the mitochondrial cytochrome c oxidase I (COI) primer set (mlCOIintF and jgHCO2198; Table A1) to achieve higher taxonomic resolution.

**Table A1:** Primer sequences used in this study.

Target Organisms (Region)	Name	Sequences (5′ → 3′)	References
Eukaryotes (18SV9)	18SV9_F	TCGTCGGCAGCGTCAGATGTGTATAA GAGACAGCCCTGCCHTTTGTACACAC	Kim et al., 2023 [96]
	18SV9_R	GTCTCGTGGGCTCGGAGATGTGTATAA GAGACAGCCTTCYGCAGGTTCACCTAC	
Animal (COI)	mlCOIintF	GGWACWGGWTGAACWGTWTAYCCYCC	Leray et al., 2013 [97]
	jgHCO2198	TAIACYTCIGGRTGICCRAARAAYCA	

For both marker sets, we used AccuPower HotStart PCR PreMix (Bioneer, Daejeon, Republic of Korea) with a total PCR reaction volume of 20 μL (DNA template 1 μL, forward primer 1 μL, reverse primer 1 μL, and distilled water 17 μL). The PCR conditions consisted of: 1 cycle of initial denaturation (94 °C, 10 min), 35 cycles of denaturation (94 °C, 1 min), annealing (50 °C, 1.5 min), extension (72 °C, 1 min), and 1 cycle of final extension (72 °C, 10 min). After the first PCR, we confirmed product size through 1.5% agarose gel electrophoresis and stored the products at −20 °C.

The second PCR was performed using KAPA HiFi HotStart ReadyMix (KAPA Biosystems, Wilmington, MA, USA) and Nextera XT Index Kit v2 (Illumina, San Diego, CA, USA) with a total reaction volume of 25 μL (first PCR product 2.5 μL, forward index 2.5 μL, reverse index 2.5 μL, KAPA mix 12.5 μL, distilled water 5 μL). The PCR conditions consisted of: 1 cycle of initial denaturation (95 °C, 3 min), 10 cycles of denaturation (95 °C, 30 s), annealing (55 °C, 30 s), extension (72 °C, 30 s), and 1 cycle of final extension (72 °C, 5 min). Subsequently, we confirmed product size through 1.5% agarose gel electrophoresis and stored the products at −20 °C.

PCR products were purified using a bead clean-up process with AMPure XP Reagent (Beckman Coulter, Indianapolis, IN, USA) and then pooled in equal concentration (10 nM) using a DeNovix QFX Fluorometer and DeNovix dsDNA Ultra High Sensitivity Assay (DeNovix, Wilmington, DE, USA) according to the manufacturer’s protocol. The generated library was stored at −20 °C until DNA sequencing.

For metabarcoding analysis, all 36 faecal samples were analyzed using the 18S V9 primers, while COI-based analysis was successfully completed for 31 samples (14 from restored areas and 17 from alternative areas). The difference in successful amplification rates between the two markers may be attributed to varying DNA quality or primer specificity. All subsequent COI-based prey composition analyses were conducted using this subset of 31 samples, while the broader taxonomic analyses using 18S V9 included all 36 samples.

### Appendix A.2. DNA Sequence Analysis

Library samples were sequenced using NGS on an Illumina iSeq platform (Illumina, San Diego, CA, USA), and data processing was performed using USEARCH (v11.0.667) [98]. Demultiplexed raw sequences (FASTQ files) were merged into single sequences, allowing a maximum of ten mismatches. Merged reads with expected errors > 1.0 were discarded after quality filtering. The remaining sequences were dereplicated and clustered into OTUs at a 97% similarity cut-off value, removing chimeric and singleton sequences.

The resulting OTU sequences were searched against the National Centre for Biotechnology Information (NCBI) database [99] using BLASTn [100]. A list of the 100 top-scoring taxa for each OTU was obtained. OTUs were provisionally identified based on identity percentages, with OTUs exhibiting ≥ 97% identity assigned at the species level, while those with 90–97% identity were assigned at the genus or family level. Subsequently, we referenced the biodiversity database of South Korea (National Institute of Biological Resources 2024 [99]) to confirm the presence of taxa within the nation’s territory. In cases where taxa were not confirmed in the reference database, we considered either higher taxonomic ranks or second-best scoring taxa. OTUs identified as *H. yangi* were considered ‘self-DNA’ and excluded from subsequent analyses. The obtained sequences were deposited in the NCBI repository under accession numbers PRJNA1284613 and PRJNA1281337.

### Appendix A.3. Ethical Approval

The research was conducted under the endangered species capture and release permit (No. 2024-1) issued by the Nakdong River Basin Environmental Office for the Yangsan Sasong Public Housing District Development Project. The permit authorized the capture and relocation of all *H. yangi* individuals found within the development area in Sasong-ri and Naesong-ri, Dongmyeon, Yangsan City, Gyeongnam Province. The permit was valid from 11 January 2024, to 15 February 2025, covering the entire research period. All procedures were conducted under expert supervision in strict compliance with the permit conditions, including immediate relocation protocols to alternative habitats and mandatory quarterly reporting requirements. The permit specifically allowed for genetic analysis for species identification purposes, which enabled the dietary analysis component of this study.

All procedures strictly followed applicable international, national, and institutional guidelines for the care and use of animals and complied with current laws of the Republic of Korea. All observations and experiments conducted in this study are in agreement with the ethical recommendations of the College of Biology and the Environment at Pusan National University. To minimize any potential impact on this endangered species, we employed exclusively non-invasive methods, collecting only faecal samples from *H. yangi* larvae without handling, restraining, or otherwise disturbing the animals. This approach ensured zero mortality or injury to the study subjects while still obtaining valuable dietary information. We did not collect adult individuals as voucher specimens, in line with the IUCN recommendation on research involving species at risk of extinction [101,102]. This study demonstrates how conservation research can generate meaningful scientific insights while adhering to the highest standards of ethical consideration for vulnerable species.

**Table A2:** Blocking primer sequences developed for this study.

	Sequence (5′ → 3′)	Template Strand	Length	Start	Stop	Tm	GC%	Length (bp)
Block_18SV9_F	GCTACTACCGATTGGATGGTTTA	Plus	23	28	50	58.05	43	72
Block_18SV9_R	TCCGCGAGGGCCGTT	Minus	15	99	85	60.76	73	

**Table A3:** Prey items detected from fecal samples of *Hynobius yangi* using 18SV9 primers (n = 36) and corresponding BLASTn results.

Primer Group	Classification	Genus + Species	Max Score	Identity (%)	Query (%)	Genbank Accession
18SV9	Benthic Invertebrates:	*Gammarus pulex*	322	98.4%	100%	AF202982.1
		*Attheyella crassa*	272	95.9%	96%	EU380307.1
		*Tubifex ignotus*	316	98.3%	100%	DQ280316.1
		*Bryocamptus pygmaeus*	303	97.7%	100%	AY627015.1
		*Heterolepidoderma kolickae*	309	98.3%	100%	OQ358136.1
		*Schmidtea mediterranea*	315	98.9%	100%	U31085.1
		*Dugesia japonica*	315	98.9%	100%	AF013153.1
	Plants:	*Nymphaea colorata*	309	98.3%	100%	LR722160.1
		*Pinus mugo* subsp. *rotundata*	313	99.4%	96%	PQ094667.1
		*Schizonepeta tenuifolia*	324	99.4%	100%	JN802671.1
		*Ajania tenuifolia*	318	98.9%	100%	OP737764.1
	Zooplankton:	*Brachionus diversicornis*	137	80.8%	100%	MK106113.1
		*Pleuroxus truncatus*	318	95.1%	98%	AM490291.1
		*Acroperus harpae*	315	94.7%	98%	AM490272.1
		*Macropelopia paranebulosa*	311	97.8%	100%	OQ808864.1
	Microalgae/Protists:	*Chlorolobion braunii*	315	99.4%	100%	KT833591.1
		*Desmodesmus communis*	316	99.4%	100%	KF864475.1
		*Colacium vesiculosum*	270	97.5%	96%	AF081592.1
		*Pinnularia* cf. *gibba*	313	99.4%	100%	EF151977.1
		*Ankistrodesmus falcatus* var. *tumidus*	315	99.4%	100%	JQ315498.1
		*Scenedesmus* sp. R4	318	99.4%	100%	KF879582.1
		*Gomphonema* sp. 12	313	99.4%	100%	KJ961663.1
		*Tritrichomonas musculus*	222	95.0%	95%	ON907819.1
		*Chilomastix* sp. IC-2014	252	99.3%	87%	KC960593.1
		*Hemolivia stellate*	230	90.8%	100%	KP881349.1
		*Lambornella* sp.	279	98.7%	100%	AF364043.1
		*Ephelota* sp. CXR06101202	145	83.4%	100%	GQ265956.1
	Amphibians:	*Pseudophryne corroboree*	239	90.6%	100%	XR_010173549.1
	Terrestrial/Semi-aquatic Invertebrates:	*Diachasma alloeum*	302	97.2%	100%	XR_003783141.1
		*Planococcus citri*	320	98.9%	100%	OX465509.1
		*Lucilia sericata*	315	98.3%	100%	XR_005232340.1
		*Physa fontinalis*	326	99.4%	99%	PP930947.1
		*Hypsibius* sp. ‘Moon 1997’	302	97.2%	100%	Z93337.1
	Unclassified/Other:	*Thulinius stephaniae*	294	99.4%	90%	AF056023.1
		uncultured eukaryote	303	97.7%	100%	AB902356.1
		uncultured Tubificina	327	99.4%	100%	AY605206.1
		*Pistocythereis* sp. QY-2003	318	98.9%	100%	AY191449.1
		*Anurofeca* sp. LAH-2003	309	98.3%	100%	AY363958.1
		uncultured Eimeriidae	111	79.3%	97%	EF024347.1
		uncultured Cercozoa	267	95.8%	97%	EF023523.1

**Table A4:** Prey items detected from fecal samples of *Hynobius yangi* using COI313 primers (n = 31) and corresponding BLASTn results.

Primer Group	Class	Genus + Species	Max Score	Identity (%)	Query (%)	Genbank Accession
COI313	Insecta	*Nemoura geei*	593	98%	92%	MK132385.1
		*Podocopida gen.* sp. BIOUG021	599	99%	92%	MG316806.1
		*Crisicoccus pini*	595	99%	92%	HM474131.1
		*Polypedilum convexum*	616	99%	92%	LC050953.1
		*Cecidomyiidae* sp.	593	98%	92%	OR173512.1
		*Psectrotanypus orientalis*	603	99%	92%	LC329249.1
		*Thienemanniella majuscula*	588	98%	92%	OM502167.1
		*Cecidomyiidae* sp. BIOUG14616-D01	52.8	100%	9%	MF698829.1
		*Micropsectra daisenensis*	604	99%	92%	LC329118.1
		*Thienemanniella majuscula*	577	97%	92%	OM502167.1
		*Bradysia* sp. BOLD-2016	63.9	100%	12%	KR443771.1
	Clitellata	*Aulodrilus pluriseta*	577	97%	92%	LT905384.1
		*Chaetogaster* sp. 10 JM-2023	636	98%	99%	OQ281718.1
		*Nais communis*	610	99%	92%	LN810253.1
		*Aulodrilus pluriseta* ABWD_17	523	98%	83%	MW703526.1
	Gastropoda	*Physella acuta*	619	98%	97%	OW483461.1
	Amphibia	*Rana dybowskii*	625	98%	98%	KM876947.1

**Table A5:** Prey items detected from fecal samples of *Hynobius yangi* using Block_18SV9 primers (n = 24) and corresponding BLASTn results.

Primer Group	Classification	Genus + Species	Max Score	Identity (%)	Query (%)	Genbank Accession
Block_ 18SV9	Benthic Invertebrates:	*Asellus aquaticus*	337	98.43%	100%	AF255701.1
		*Aspidiophorus* sp. n. MK-2019	315	98.86%	100%	MN496181.1
		*Magallana gigas*	331	99.45%	100%	AB064942.1
		*Nemoura cinerea*	318	98.34%	100%	EF622738.1
		*Steginoporella truncata*	294	97.14%	100%	FJ009098.1
		*Stylaria* sp. ‘Aguinaldo, 1997’	327	99.44%	100%	U95946.1
	Plants:	*Artemisia scoparia*	318	98.88%	100%	OP739248.1
		*Cissus quadrangularis*	302	97.19%	100%	MW583102.1
		*Cornus florida*	318	98.88%	100%	XR_009476696.1
		*Diplazium hewittii*	307	97.75%	100%	PQ144161.1
		*Equisetum robustum*	329	100.00%	100%	X78890.1
		*Scutellaria heterophylla*	318	98.88%	100%	MW396605.1
	Zooplankton:	*Bryocamptus pygmaeus*	309	98.30%	100%	AY627015.1
		*Cyclops* sp. UK-RJH-2004	307	98.30%	100%	AY626998.1
		*Eucyclops serrulatus*	303	97.73%	100%	L81940.2
		*Oithona* sp. Roadstead of Brest	313	98.86%	100%	KX364950.1
	Microalgae/ Protists:	*Amanita inzengae*	315	98.86%	100%	PV275608.1
		*Ankistrodesmus* sp. NDem 9/21 T-6w	315	99.42%	100%	AY846374.1
		*Aulacoseira ambigua*	309	98.84%	100%	X85404.2
		*Aulacoseira baicalensis*	292	0.9711	100%	AJ535185.1
		*Bodo saltans*	285	98.75%	100%	AY490234.1
		*Cercomonas* sp.	316	98.87%	100%	PP174297.1
		*Chilomastix caulleryi*	252	99.28%	87%	AB600326.1
		*Chloromonas insignis*	322	100.00%	100%	JQ315775.1
		*Chlorophyceae* sp. DC-11	316	99.43%	100%	JF834169.1
		*Choricystis limnetica*	320	0.9943	100%	MT423986.1
		*Cutaneotrichosporon debeurmannianum*	320	99.43%	100%	AB035584.1
		*Desmodesmus abundans*	322	100.00%	100%	OR168645.1
		*Desmodesmus tropicus*	311	98.85%	100%	OR576705.1
		*Euglena agilis*	296	98.80%	96%	KU941987.1
		*Eustigmatophyceae* sp. RaA-2019f	274	100.00%	88%	MN389516.1
		*Eustigmatophyceae* sp. Tow 9/21 P-2w	285	97.04%	100%	KF757253.1
		*Gonium pectorale*	307	98.84%	100%	MF677989.1
		*Leonella granifera*	309	98.84%	100%	KF751921.1
		*Nadsonia starkeyi-henricii*	305	0.994	95%	KY105365.1
		*Nannochloropsis oceanica*	305	99.40%	100%	PQ645491.1
		*Nitzschia* sp. (in: diatoms)	311	98.85%	100%	OR922684.1
		*Pinnularia* cf. *gibba*	307	98.84%	100%	EF151977.1
		*Polymyxa graminis*	318	99.43%	100%	AM110920.1
		*Pseudogomphonema* sp. p382	303	99.40%	96%	AJ535152.1
		*Rhogostoma schuessleri*	318	99.43%	100%	HQ121430.1
		*Rhogostoma* sp. CCAP 1966/4	315	99.42%	100%	HQ121431.1
		*Scenedesmus quadricauda*	316	99.43%	100%	KT778085.1
		*Spumella bureschii*	311	98.85%	100%	MZ420278.1
		*Stephanodiscus hantzschii*	305	98.28%	100%	AB831892.1
		*Tetratrichomonas prowazeki*	261	99.31%	96%	OK584282.1
		*Volvox carteri f. nagariensis*	302	98.26%	100%	X53904.1
	Amphibians:	*Pseudophryne corroboree*	311	97.78%	100%	XR_010173541.1
		*Rana temporaria*	316	98.33%	100%	XR_005744675.1
	Terrestrial/ Semi-aquatic Invertebrates:	*Eretmapodites intermedius*	326	99.44%	100%	OM350289.1
		*Kogia breviceps*	329	99.45%	100%	XR_009333359.1
		*Nurscia albofasciata*	320	99.43%	100%	KR074017.1
		*Proisotomodes bipunctatus*	320	98.88%	100%	PP864448.1
	Unclassified/Other:	*Anurofeca* sp. LAH-2003	315	0.9886	100%	AY363958.1
		*Aphelidiaceae* sp.	294	97.67%	100%	OQ702869.1
		uncultured alveolate	298	99.39%	98%	EU162625.1
		uncultured Ascomycota	320	99.43%	100%	AM901900.1
		uncultured Basidiomycota	318	99.43%	100%	AM902048.1
		uncultured dinoflagellate	313	0.9831	100%	AY179990.1
		uncultured Eimeriidae	296	99.39%	96%	EF023637.1
		uncultured eukaryote	283	99.35%	94%	KY690246.1
		uncultured fungus	309	98.30%	100%	KF800647.1
		uncultured *Hydrurus* sp.	302	98.26%	99%	MG674912.1
		uncultured marine picoeukaryote	318	99.43%	100%	FR874598.1
		uncultured Pyronemataceae	303	97.73%	100%	FJ553250.1
		uncultured soil eukaryote	320	99.43%	100%	MK946212.1
		uncultured Tubificina	311	97.78%	100%	AY605206.1

**Table A6:** Summary of prey taxa detected across habitat types using three primer approaches. FOO: Frequency of Occurrence.

Primer Set	Habitat Type	Sample Size	Total Taxa	Dominant Groups (>20% FOO)
18S V9	Restored	18	32	Zooplankton, Microalgae/Protists, Unclassified
18S V9	Alternative	18	35	Benthic Invertebrates, Zooplankton, Microalgae/Protists
COI313	Restored	14	12	Chironomidae, Psychodidae, Nematocera
COI313	Alternative	17	15	Perlidae, Tubificidae, Chironomidae
Block_18SV9	Restored	12	28	Microalgae/Protists, Zooplankton, Unclassified
Block_18SV9	Alternative	12	31	Benthic Invertebrates, Microalgae/Protists, Zooplankton
